# Dorsal hippocampal astrocytes mediate the development of heroin withdrawal-enhanced fear learning

**DOI:** 10.1007/s00213-024-06562-4

**Published:** 2024-02-24

**Authors:** Shveta V. Parekh, Lydia O. Adams, Gillian A. Barkell, Jacqueline E. Paniccia, Kathryn J. Reissner, Donald T. Lysle

**Affiliations:** Department of Psychology and Neuroscience, University of North Carolina at Chapel Hill, CB#3720, Chapel Hill, NC 27599-3270 USA

**Keywords:** Astrocyte, Post-traumatic stress disorder (PTSD), Opioid use disorder (OUD), Dorsal hippocampus (DH), G_i_ signaling

## Abstract

There is a significant co-occurrence of opioid use disorder (OUD) and post-traumatic stress disorder (PTSD) in clinical populations. However, the neurobiological mechanisms linking chronic opioid use, withdrawal, and the development of PTSD are poorly understood. Our previous research has shown that proinflammatory cytokines, expressed primarily by astrocytes in the dorsal hippocampus (DH), play a role in the development of heroin withdrawal-enhanced fear learning (HW-EFL), an animal model of PTSD-OUD comorbidity. Given the role of astrocytes in memory, fear learning, and opioid use, our experiments aimed to investigate their involvement in HW-EFL. Experiment 1 examined the effect of withdrawal from chronic heroin administration on GFAP surface area and volume, and identified increased surface area and volume of GFAP immunoreactivity in the dentate gyrus (DG) following 24-hour heroin withdrawal. Experiment 2 examined astrocyte morphology and synaptic interactions at the 24-hour withdrawal timepoint using an astroglial membrane-bound GFP (AAV5-GfaABC1D-lck-GFP). Although we did not detect significant changes in surface area and volume of GfaABC1D-Lck-GFP labelled astrocytes, we did observe a significant increase in the colocalization of astrocyte membranes with PSD-95 (postsynaptic density protein 95) in the DG. Experiment 3 tested if stimulating astroglial G_i_ signaling in the DH alters HW-EFL, and our results demonstrate this manipulation attenuates HW-EFL. Collectively, these findings contribute to our current understanding of the effects of heroin withdrawal on astrocytes and support the involvement of astrocytes in the comorbid relationship between opioid use and anxiety disorders.

## Introduction

Post-traumatic stress disorder (PTSD), a devastating psychological disorder, commonly co-occurs with other psychiatric disorders, such as substance use disorder. In fact, the prevalence of opioid use disorder (OUD) and PTSD comorbidity is as high as 50% (Roberts et al. 2015). Notably, when compared to individuals with either disorder alone, those with PTSD and co-occurring heroin use disorder have longer addiction durations, higher rates of suicide, and worsened clinical outcomes across occupational, financial, and social domains (McDevitt-Murphy et al. 2010; Tate et al. 2007). In turn, elucidating the underlying neurobiological mechanisms of this comorbidity represents an important area of research, as these mechanisms provide an avenue for treatment development and improved outcomes in this clinical population.

To examine the comorbidity between PTSD and opioid use disorders, our laboratory investigates heroin use and withdrawal in conjunction with stress-enhanced fear learning (SEFL) using a model known as heroin-withdrawal enhanced fear learning (HW-EFL). This model involves 10 days of chronic escalating heroin administration followed by a mild foot shock during withdrawal. Strikingly, this prolonged opioid exposure and subsequent withdrawal sensitizes the animals to display hyperarousal and hyper-reactivity in response to the mild foot shock stressor. We hypothesize that this increased vulnerability to stress, which models clinical PTSD-OUD comorbidity, results from neurobiological changes following heroin use and withdrawal. These neurobiological changes may increase an individual’s susceptibility to developing PTSD symptomology, such as enhanced fear learning, vulnerability to future stressors, and hyperarousal (Schell et al. 2004). One such neurobiological change that may underlie this increased prevalence of PTSD symptomology in opioid use disorder is immune dysregulation. Indeed, altered cytokine expression is observed in both human and animal models (Lashkarizadeh et al. 2016; O’Sullivan et al. 2019; Wang et al. 2018) across multiple anxiety disorders, including PTSD (Gola et al. 2013; Hori and Kim 2019; Kim et al. 2020). In line with these findings, our lab has observed increased expression of the proinflammatory cytokines interleukin-1 beta (IL-1β) and tumor necrosis factor alpha (TNF-α) in the dorsal hippocampus (DH) following heroin withdrawal (Parekh et al. 2020, 2021). Further, blockade of these enhanced cytokine levels attenuates the development of HW-EFL, our animal model of comorbid PTSD-OUD (Parekh et al. 2020, 2021). These findings support the notion that PTSD-OUD comorbidity may have a neuroimmune basis.

There is growing evidence that astrocytes mediate many neuroimmune interactions in the brain, and thus may be critical to understanding the neurobiological mechanisms driving the relationship between opioid use, cytokine signaling, and enhanced fear learning and anxiety. Interestingly, we have demonstrated that astrocytes play a critical role in the enhanced cytokine signaling of proinflammatory cytokines (IL-1β and TNF-α) in the dorsal hippocampus following heroin withdrawal, supporting the notion that astrocytes may functionally promote this comorbidity (Jones et al. 2018a; Parekh et al. 2020, 2021). Astrocytes may participate in this comorbidity by directly influencing neuronal function through the release of these cytokines, as well as other gliotransmitters (Haydon and Carmignoto 2006; Lacagnina et al. 2017; Santello and Volterra 2012). Furthermore, there is evidence linking glial-mediated mechanisms to the prevention of maladaptive stress responses in rodent models, providing further support for their essential role in fear-learning processes (Ben Menachem-Zidon et al. 2011; Levkovitz et al. 2015; Xia et al. 2013). Our laboratory has also demonstrated that chronic opioid use and withdrawal is associated with enhanced astrocyte reactivity, indicated by increased immunoreactivity of glial fibrillary acidic protein (GFAP), a filament protein expressed in astrocytes (Parekh et al. 2020). This suggests that opioid use and withdrawal could potentially induce changes in the reactivity and functioning of astrocytes. Collectively, these studies support the idea that astrocytes may mediate PTSD-OUD comorbidity following heroin use and withdrawal.

To study astrocyte reactivity in response to heroin use and withdrawal, we examined changes in the surface area and volume of GFAP, as increased GFAP immunoreactivity is generally viewed as an index of gliosis (Eng and Ghirnikar 1994; Yang and Wang 2015). Further, we examined changes in astrocyte morphology induced by heroin withdrawal. Studies to date have used GFAP to examine opioid-induced alterations in hippocampal astrocytes (Song and Zhao 2001). However, GFAP constitutes only about 15% of astrocytes’ total volume, providing only a partial or limited image of astrocyte morphology (Benediktsson et al. 2005). Therefore, GFAP cannot provide information regarding how the fine, distal processes of astrocytes interact with synapses, and how they are altered during heroin withdrawal (Scofield et al. 2016). We conducted an in-depth analysis of individual astrocytes and colocalization with post-synaptic markers using the membrane-associated AAV5-GfaABC1D-Lck-GFP construct. This method uses high-resolution confocal microscopy and advanced software analysis to reliably visualize individual astrocytes in rich detail, including their fine peripheral processes, to quantify surface area, volume, and colocalization with post-synaptic markers throughout a 3-dimensional reconstruction (Jones et al. 2018a; Testen et al. 2020). We chose to examine these morphological changes, as they could provide further insight into how astrocytes transform during withdrawal and their involvement in subsequent fear learning. Finally, to investigate the function of astrocytes during enhanced fear learning, we used astroglial-expressing designer receptors exclusively activated by designer drugs (DREADDs) to selectively manipulate dorsal hippocampal astroglial G_i_ signaling during enhanced fear learning. The G_i_-DREADD was chosen to selectively activate G_i_ signaling pathways in astrocytes in the dorsal hippocampus, which our laboratory has previously demonstrated to attenuate stress-enhanced fear learning, although has yet to examine in the context of HW-EFL (Jones et al. 2018b). Further, activation of G_i_ pathways in astrocytes has been previously shown to attenuate intracellular calcium transients triggered by LPS treatment and reduce the inflammatory phenotype of astrocytes (Kim et al. 2021). In turn, by stimulating this signaling pathway in astrocytes, we may potentially counteract the enhanced release of inflammatory cytokines during and following heroin administration and withdrawal that we have established to be mechanistically important in the development of enhanced fear learning.

In turn, the current studies test the hypothesis that chronic heroin and withdrawal is capable of inducing alterations in DH GFAP, astrocyte morphology, astrocyte-neuron interactions, and if these changes may play a role to the development of future enhanced fear learning. We chose to examine the 24-hour heroin withdrawal timepoint in these experiments due to previous findings demonstrating astrocyte-derived IL-1β and TNF-α immunoreactivity in the DG at this time point, which we have identified to be mechanistically implicated in the development of heroin-withdrawal enhanced fear learning. In further support of the 24-hour withdrawal timepoint, we have also observed increased GFAP immunoreactivity in the DG at this time point. The results from these studies significantly contribute to our present understanding of the effects of heroin withdrawal on astrocytes and provide insight into the comorbid relationship of opioid use and anxiety disorders.

## Materials and methods

### Animals

Adult male Sprague Dawley rats (225–250 g, Charles River Laboratories, Raleigh, NC) were individually housed under a reversed 12-h light-dark cycle. Rats were given ad libitum access to food and water and were regularly handled throughout experimentation. All procedures were conducted with approval from the University of North Carolina at Chapel Hill Institutional Animal Care and Use Committee.

### Stereotaxic surgery

The surgical procedures have been described previously at length (Paniccia et al. 2018; Testen et al. 2020). For all surgical procedures, animals were anesthetized with a 1 ml/kg intraperitoneal injection of 9:1 (vol/vol) ketamine hydrochloride (100 mg/mL) mixed with xylazine (100 mg/mL). In Experiment 2, animals received stereotaxic surgery to infuse AAV5-GfaABC1D-Lck-GFP (0.7 µL/hemisphere) bilaterally into the DH (AP -3.4 mm, ML ± 3.1 mm, DV -3.2 mm, relative to bregma, 15° angle laterally). Animals were given 2 weeks to recover from surgery. Additionally, Experiment 1 utilized tissue from Experiment 2 (stained with primary antibodies for GFAP) to conduct confocal analysis of GFAP surface area and volume. In Experiment 3, animals received stereotaxic surgery to infuse AAV8-GFAP-hM4D(G_i_)-mCherry (0.7 µL/hemisphere) bilaterally into the DH (AP -3.4 mm, ML ± 3.1 mm, DV -3.2 mm, relative to bregma, 15° angle laterally) and were given 2 weeks to recover.

### Drug administration

Heroin (diacetylmorphine hydrochloride, NIDA Drug Supply Program, Bethesda, MD, USA) was dissolved in sterile 0.9% saline to produce 1.0, 2.5, 5.0, 7.5, or 10.0-mg/mL solutions and stored at 4 °C until time of injection. In all experiments, the administration schedule of chronic escalating heroin (which has been previously described (Parekh et al. 2020) was 10 days of three subcutaneous injections over 24-hour periods (at 15:00, 21:00, and 9:00 the next day, UTC-05:00) and a dose increase every other day. Weight was measured at each time point to determine withdrawal-induced weight change. This effect was replicated as per previous studies. In Experiment 3, the AAV8-GFAP-hM4D(G_i_)-mCherry DREADDs ligand, clozapine-N-oxide (CNO; National Institutes of Health, Bethesda, MD) was dissolved in a vehicle of 0.9% sterile saline with 0.5% dimethyl sulfoxide (DMSO), and CNO (3 mg/kg, s.c.) was administered 0, 24, and 48 h following the last injection of heroin or saline. These timepoints were chosen for CNO administration, as we have previously identified causally relevant, astrocyte-derived pro-inflammatory cytokines to be elevated at these timepoints (Parekh et al. 2020, 2021). Therefore, we hypothesized that targeting an anti-inflammatory pathway in astrocytes at timepoints where astrocyte-derived inflammation is most significantly heightened will be most effective in attenuating HW-EFL, which we postulate is driven by an astrocytic inflammatory mechanism.

### Chronic heroin and withdrawal-enhanced fear learning

To examine the effect of astrocyte G_i_-DREADD stimulation on fear learning (Experiment 3), the HW-EFL paradigm was conducted. This procedure has been previously described at length (Parekh et al. 2020). Briefly, animals were randomly assigned to drug (heroin or saline) treatment. Animals undergo chronic escalating heroin administration and withdrawal in their home cage. Seven days after the start of withdrawal, animals were placed into a novel context for 15 min of habituation. On Day 8, animals were placed into the same context for a single scrambled foot shock (1 mA, 1s) at 3 min, 12 s. On days 9, 10, 15, 22 (Test Days 1, 2, 7, and 14) animals were placed into the same context for 8 min, 32 s and behavior was recorded to measure freezing behavior, a measure of learned fear. Ethovision XT video tracking software (Noldus Information Technology Inc.) was used to analyze freezing behavior. The activity analysis feature (Activity Threshold = 10) was used and described previously (Jones et al. 2018a, b). This feature detects the entire arena and asks what percentage of pixels in the entire arena are changing or the same. To calculate the percent of time each animal was inactive during each contextual fear test and at baseline we used the % of pixels that were inactive through the activity analysis feature in Ethovision XT.

### Tissue collection and histology

Animals were sacrificed by transcardial perfusion 24-hours into withdrawal (Experiments 1 and 2), or after the completion of HW-EFL (Experiment 3). Animals perfused 24-hours following the last injection are considered to be in the 24-hour withdrawal group. Animals were terminally anesthetized with 9:1 (vol/vol) ketamine hydrochloride (100 mg/mL) mixed with xylazine (100 mg/mL), and transcardially perfused with ice-cold phosphate buffer (PB; pH = 7.4) followed by 4% paraformaldehyde in 0.1 M PB. Brains were extracted and post-fixed in 4% paraformaldehyde for 6 h, and used 30% sucrose with 0.1% sodium azide for cryoprotection at 4 °C. Once the brains were saturated with sucrose, brains were cut into 40-µm coronal sections on a cryostat (Leica CM 3050 S, Leica Microsystems, Buffalo Grove, IL, USA).

### Confocal microscopy and image analysis

Both Experiment 1 and Experiment 2 utilized tissue stained with the primary antibodies mouse anti-PSD-95 (1:500, monoclonal, 6G6-1C9, Invitrogen) and rabbit anti-GFAP (1:500, Z0334, Agilent Dako), as well as the secondary antibodies goat anti-mouse AlexaFluor-594 (1:1000) and goat anti-rabbit AlexaFluor-647 (1:1000) for visualization. Tissue was mounted onto SuperFrost Plus slides (ThermoFisher Scientific) and coverslipped using Vectashield Hardset Antifade mounting medium (H-1400, Vector Laboratories). All images were acquired by an experimenter blind to treatment group using a Zeiss LSM800 laser-scanning confocal microscope with 405 nm, 488 nm, 561 nm, and 640 nm laser lines and ZEN Blue software suite (Zeiss). Z-stacks were acquired using a 63x oil-immersive objective, 1024 × 1024 frame size, 16-bit resolution, frame average of 4, and 0.8 μm step size. In Experiment 1, eight Z-stacks were taken per subregion, bilaterally per animal. AutoQuant X3 was used to deconvolve raw images and output files were directly exported to the Bitplane Imaris software suite. GFAP-positive astrocytes were isolated using the surface rendering software of the Imaris software. The surface was created by filtering out the other stains until GFAP was isolated in the channel, and then manipulating the intensity so background signal could be taken out. The GFAP channel was used to determine the surface area and volume data of GFAP extracted from the surface. Experiment 2 analyzed morphological changes in astrocytes using the AAV5-GfaABC1D-Lck-GFP construct. The confocal acquisition, and image analysis for astrocyte morphology and PSD-95 colocalization has been described at length (see (Testen et al. 2020). Eight Z-stacks were taken in the DG, bilaterally per animal. AutoQuant X3 was used to deconvolve raw images and output files were directly exported to the Bitplane Imaris software suite. Individual Lck-GFP-positive astrocytes were isolated using the surface rendering function of the Imaris software. Background signal was subtracted out and surface area and volume data extracted from the surface. The isolated GFP channel was the designated ROI for colocalization analysis of Lck-GFP and PSD-95 signal above a manually detected threshold.

### Statistical analysis

In Experiment 1, planned comparisons using unpaired, two-tailed Student’s t-tests were used to determine whether drug (heroin or saline) treatment altered surface area and volume of GFAP-positive astrocytes at the 24-hour withdrawal timepoint. In Experiment 2, planned comparisons using unpaired, two-tailed Student’s t-tests were used to determine whether drug (heroin or saline) treatment altered surface area, volume, and PSD-95 colocalization of Lck-GFP-positive astrocytes at the 24-hour withdrawal timepoint. In Experiment 3, a 2 (heroin, saline) x 2 (CNO, saline) x 4 (test day) repeated measures ANOVA was used to examine effects of astroglial G_i_ signaling stimulation on heroin withdrawal-enhanced fear learning, indicated by levels of freezing. All statistical tests were conducted using Statistical Package for the Social Sciences (IBM SPSS).

## Results

**Fig. 1:**
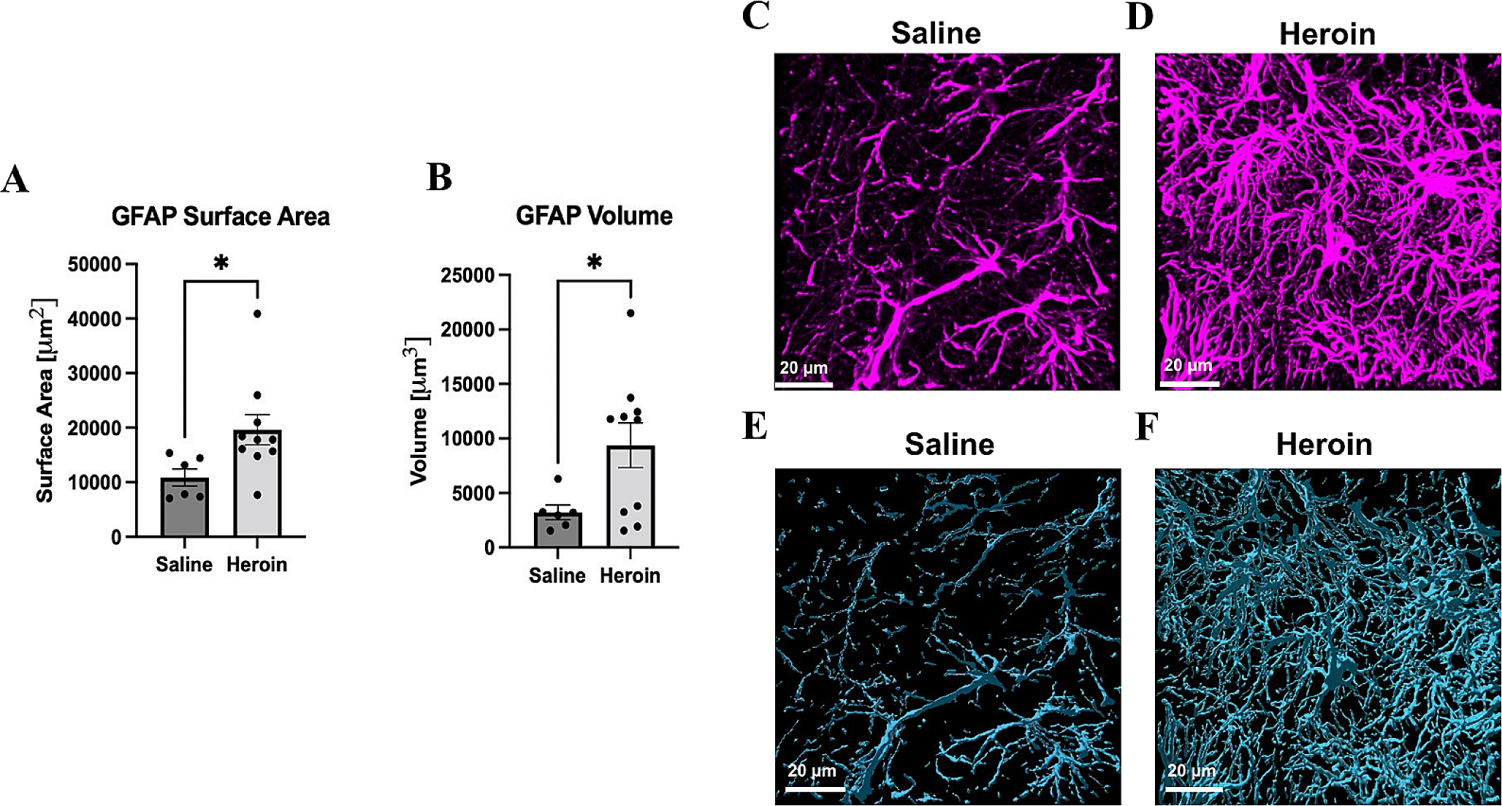
*Chronic Heroin Administration and 24-hour Withdrawal Increases GFAP Surface Area and Volume in the Dentate Gyrus.* There were significant changes in GFAP surface area (*N* = 16 total animals, *n* = 6–10 animals per treatment, eight Z-stacks were taken per region, bilaterally per animal) (**A**) and volume (*N* = 16 total animals, *n* = 6–10 animals per treatment, eight Z-stacks were taken per region, bilaterally per animal) (**B**) following 24-hour heroin withdrawal in the dentate gyrus. Representative 63x oil-immersion Z-stacks of the isolated GFAP in saline animals (**C**) relative to the heroin withdrawn animals (**D**). Representative 63x oil-immersion Z-stacks of isolated GFAP and surface for saline animals (**E**) and heroin withdrawn animals (**F**). The surface building feature of Bitplane Imaris was used to create a surface surrounding the GFAP signal to reduce background noise and isolate GFAP to obtain morphometric data. *, statistically significant (*p* < .05) difference relative to respective control. Error bars indicate SEM

*Experiment 1: Withdrawal from Chronic Heroin Administration Increases GFAP Surface Area and Volume in the Dentate Gyrus (*Fig. 1*)*.

Experiment 1 determined the consequence of chronic heroin administration and withdrawal on GFAP surface area and volume. Chronic heroin administration and 24-hour withdrawal significantly altered the surface area and volume of GFAP in the DG of the DH. There was a significant increase in the surface area of GFAP-positive staining in animals that underwent withdrawal from chronic heroin administration (*t*_*(*14)_ = 2.287, *p* = .038) relative to saline controls (Fig. 1A). Additionally, withdrawal from chronic heroin administration animals had significantly increased GFAP volume (*t*_(14)_ = 2.15, *p* = .041) in comparison to their saline counterparts (Fig. 1B). These results indicate that withdrawal from chronic heroin administration increases GFAP immunoreactivity, which is a measure of GFAP expression. These data represent all GFAP positive expression. GFAP is a well-established marker of astrocytic reactivity, and therefore these data indicate a change in astrocyte reactivity rather than a morphological change such as the number of astrocyte processes.

**Fig. 2:**
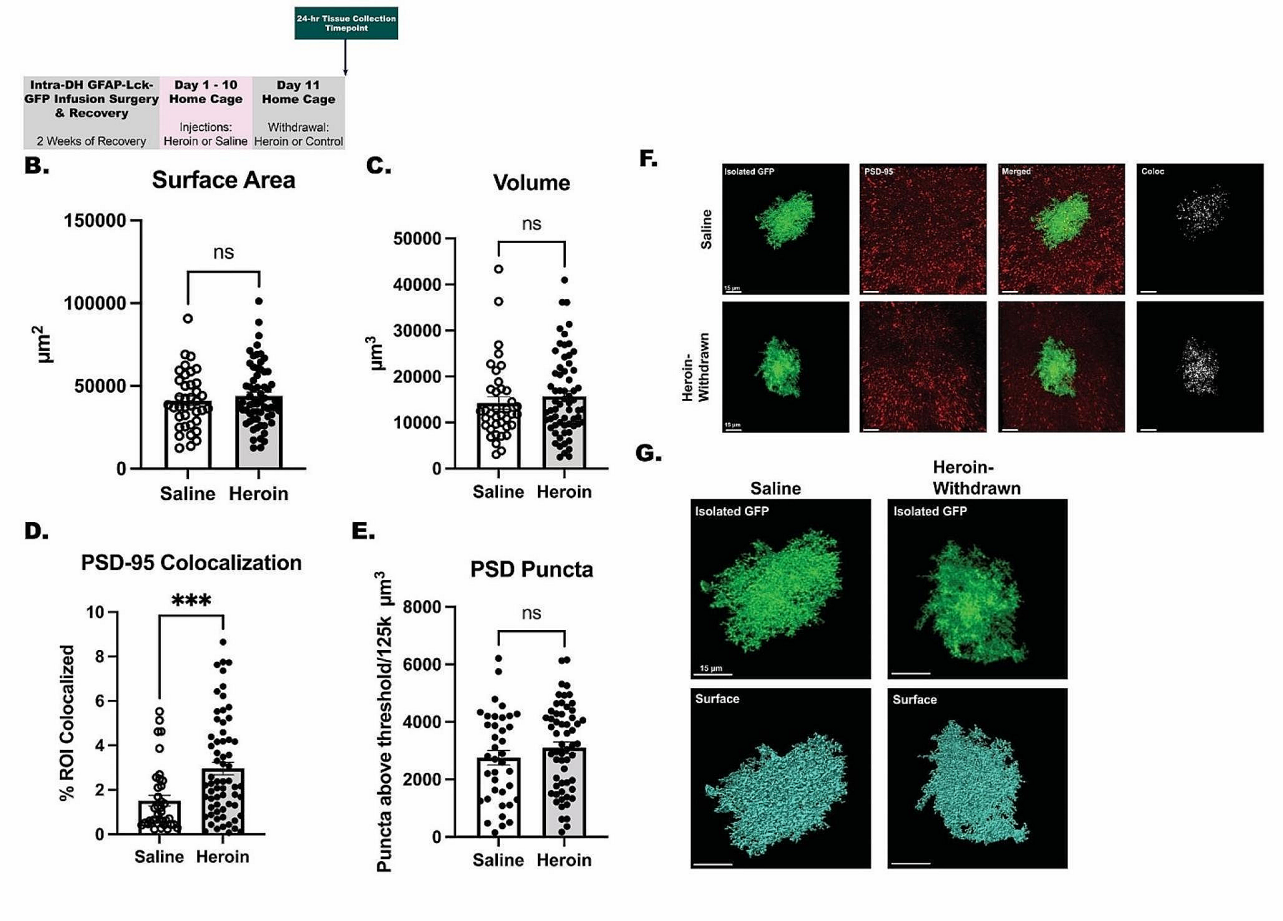
Withdrawal from Chronic Heroin Administration Does Not Alter Morphology of LCK-GFP-positive Astrocytes, but Does Increase Astrocyte-Neuron Interactions in the Dentate Gyrus. Experimental timeline **(A).** There were no significant changes in astrocyte surface area (*N* = 102 total LCK-positive cells, *n* = 38–64 LCK-positive cells from across 8–9 animals per treatment) **(B)** or volume (*N* = 102 total LCK-positive cells, *n* = 38–64 LCK-positive cells taken from 8–9 animals per treatment) **(C)** of Lck-GFP following heroin withdrawal within the dentate gyrus. Heroin withdrawal significantly increased astrocyte colocalization with PSD-95 (*N* = 102 LCK-positive cells, *n* = 38–64 LCK-positive cells from across 8–9 animals per treatment) **(D)** in heroin withdrawn animals relative to saline counterparts. Heroin withdrawal did not alter the overall number of PSD-95 puncta in the DG (*N* = 102 LCK-positive cells, *n* = 38–64 LCK-positive cells from across 8–9 animals per treatment) **(E)**. Representative 63x oil-immersion Z-stacks of the isolated GFP, PSD-95 (Alexa Fluor-594), and generated colocalization channel **(F).** Representative 63x oil-immersion Z-stacks of isolated GFP and surface. The surface building feature of Bitplane Imaris was used to create a surface surrounding the Lck-GFP signal to isolate individual cells and obtain morphometric data (**G**). *, statistically significant (*p* < .05) difference relative to respective control. Error bars indicate SEM

*Experiment 2: Withdrawal from Chronic Heroin Administration Does Not Alter Morphology of Lck-GFP-positive Astrocytes, but Does Increase Astrocyte-Neuron Colocalization in the Dentate Gyrus (*Fig. 2*)*.

Experiment 2 examined astrocyte morphology and synaptic interactions at the 24-hour withdrawal timepoint. Chronic heroin administration and 24-hour withdrawal did not alter astrocyte morphology in the DG of DH. There was no difference in astrocyte surface area (*t*_(100)_ = 0.7946, *p* > .05) or volume (*t*_(100)_ = 0.8440, *p* > .05) relative to saline controls (Fig. 2B and C). Interestingly, a history of heroin administration and withdrawal increased colocalization of astrocytes with PSD-95 (*t*_(100)_ = 3.505, *p* < .001) (Fig. 2D) in the dentate gyrus, without effect on PSD-95 positive puncta (*t*_(100)_ = 1.084, *p* > .05) (Fig. 2E). These results indicate that withdrawal from chronic heroin administration does not alter astrocyte morphology but does play a role in astrocyte-neuron interactions in the DG of the DH.

**Fig. 3:**
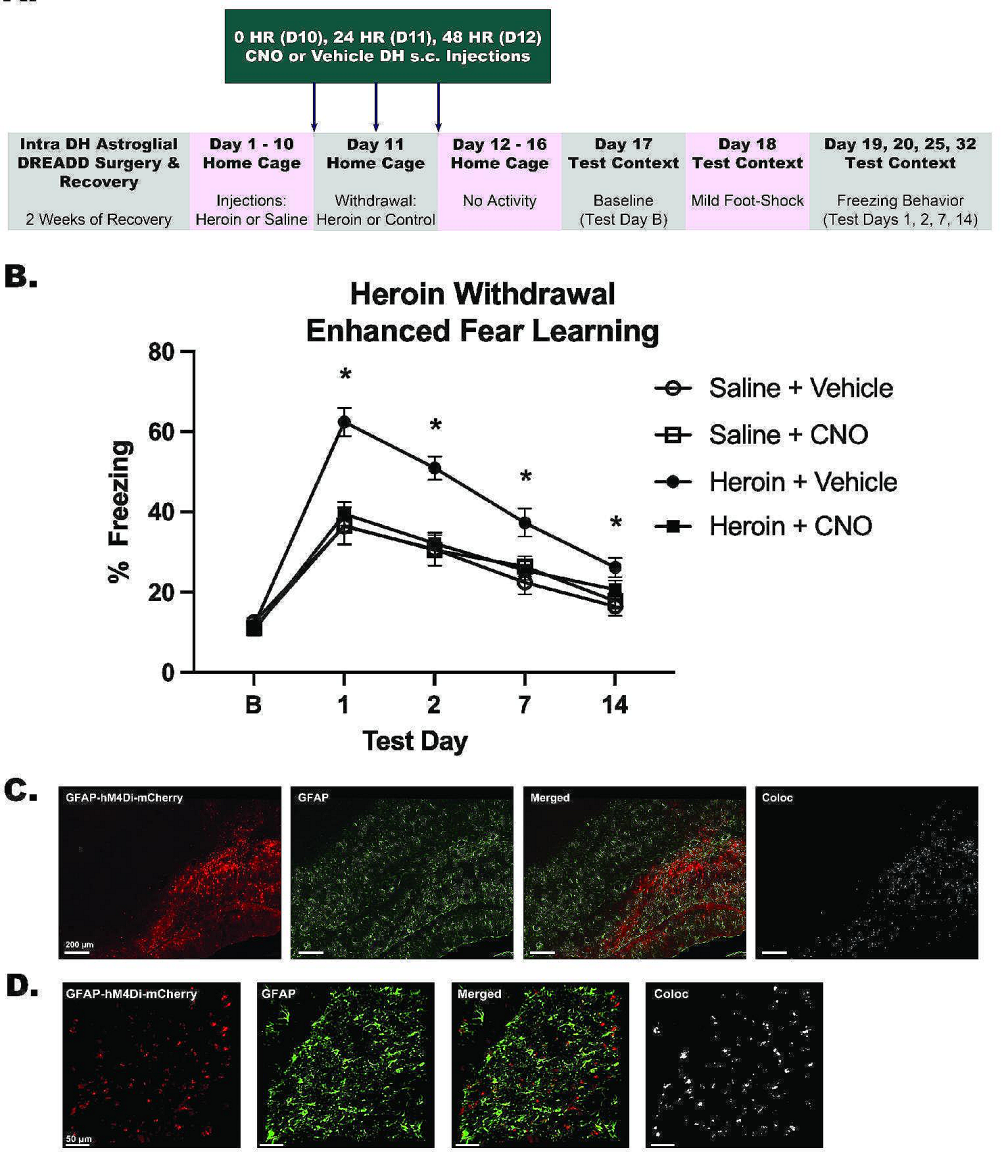
Stimulation of Astroglial G_i_ Signaling in the Dentate Gyrus Attenuates Heroin Withdrawal-Enhanced Fear Learning. Experimental Timeline **(A).** Stimulation of astroglial G_i_ signaling attenuated HW-EFL (*N* = 37, *n* = 9–10 animals) **(B).** Representative confocal 10X tile image from a heroin-withdrawn animal depicts robust mCherry expression and spread throughout the DG of DH, and demonstrates the mCherry tag is colocalized with astroglial marker GFAP (AlexaFluor-488) **(C).** Representative 20X images from a saline animal demonstrating that mCherry fluorescence is colocalized with astroglial marker, GFAP (AlexaFluor-488) **(D).** Background signal was subtracted out using Bitplane Imaris Software. *, statistically significant (*p* < .05) difference relative to respective control. Error bars indicate SEM

*Experiment 3: Stimulation of Astroglial G*_*i*_*Signaling in the Dentate Gyrus Attenuates Heroin Withdrawal-Enhanced Fear Learning (*Fig. 3*)*.

Experiment 3 tested if stimulating astroglial G_i_ signaling attenuates HW-EFL due to the pathway’s inhibitory role in neuroinflammation, as we have previously identified neuroinflammation to be mechanistically critical in the development of enhanced fear learning. Stimulation of astroglial G_i_ signaling prevented the development of heroin withdrawal-enhanced fear learning following withdrawal from chronic heroin administration (Fig. 3B). There was no effect of heroin treatment or CNO infusion on baseline contextual freezing (*F*_(3,23)_ = 0.323, *p* = .809), indicating that there is no generalized fear to the novel context. A 2 × 2 × 4 repeated measures ANOVA revealed a significant main effect of heroin treatment (*F*_(1,33)_ = 19.025, *p* <. 001) and a significant main effect of CNO treatment (*F*_(1,33)_ = 9.286, *p* = .005). These main effects of heroin treatment and CNO treatment were on contextual freezing. There was also a significant effect of test day (*F*_(3,99)_ = 56.360, *p* < .001), indicating that conditioned freezing behavior diminished over time. Importantly, there was a significant heroin treatment by CNO treatment interaction (*F*_(1,33)_ = 13.252, *p* = .001). Tukey’s post hoc comparisons revealed heroin withdrawn, vehicle-treated animals exhibited significantly higher freezing behavior compared to animals that were saline controls and vehicle-treated (*p* < .05), replicating HW-EFL. Heroin-withdrawn, CNO-treated animals exhibited significantly less freezing than withdrawn animals that received vehicle (*p* < .05). Furthermore, heroin-withdrawn, CNO-treated animals exhibited a comparable amount of freezing behavior (no significant difference) to both saline control groups (*p* > .05). These results indicate that stimulation of astroglial G_i_ signaling during heroin withdrawal prevented future enhanced fear learning.

## Discussion

The current studies demonstrate that DH astrocytes undergo changes in their reactivity, cellular interactions, and intracellular signaling during chronic heroin exposure and withdrawal, and that these changes may play a role in enhanced fear learning. We show that chronic heroin exposure and withdrawal is associated with alterations in astrocyte reactivity, including increased surface area and volume of GFAP immunoreactivity in the DG. However, this effect is not maintained when surface area and volume of GFP from the GfaABC1D-Lck-GFP viral construct is analyzed, indicating that astrocyte morphology did not increase concurrently with astrocyte reactivity. Along with this enhanced reactivity, we also observed an increase in astrocyte-neuron interactions, signified by increased colocalization of PSD-95 and GfaABC1D-Lck-GFP at the 24-hour withdrawal time point. Critically, we also see that manipulating astrocytic intracellular signaling has functional consequences on the maladaptive behavioral changes that occur following chronic heroin and withdrawal. Specifically, we show that stimulating G_i_ signaling in DH astrocytes during withdrawal blocked the development of heroin withdrawal-enhanced fear learning. These findings provide potential new evidence that heroin withdrawal-induced astrocytic modifications may play a role in the development of future enhanced fear learning and could be a potential mechanism by which opioids and opioid withdrawal can elicit fear- and arousal-related features of PTSD symptomatology.

The present findings illustrate that enhanced astrocyte reactivity is associated with withdrawal from chronic heroin administration and may also be involved in HW-EFL. Specifically, we found that exposure to withdrawal from chronic heroin administration increases the volume and surface area of GFAP reactivity in the DG using confocal microscopy to create a 3D-reconstruction of GFAP-positive staining in the astrocytes. This complements our previous finding, which showed a percent area increase in DG-GFAP immunoreactivity at the 24-hour heroin withdrawal timepoint (Parekh et al. 2020). Additionally, we chose to focus on the 24-hour timepoint as our previous findings have supported the notion that heroin withdrawal, rather than heroin administration, drives these observed increases in GFAP expression as GFAP immunoreactivity was not increased simply after the administration of heroin (Parekh et al. 2020). The increased GFAP surface area and volume observed in the present study is indicative of an activated astrocyte profile, as reactive astrocytes tend to have upregulated levels of GFAP, leading to an increased number of GFAP-positive cells (Beitner-Johnson et al. 1993).

In further support of these observed effects on astrocyte reactivity, it has been previously identified that repeated opioid administration induces a pro-inflammatory state in astrocytes, including upregulated activation markers (Hutchinson et al. 2011). One hallmark of such astrocytic activation is the increased release of pro-inflammatory cytokines, which our lab has also demonstrated to be elevated following heroin administration and withdrawal (Parekh et al. 2020, 2021). In turn, these changes in reactivity markers may be associated with enhanced fear learning by inducing pro-inflammatory cytokine release, which influences anxiety-like behaviors and synaptic plasticity (Cao et al. 2013; Fellin et al. 2004; Shigetomi et al. 2008), and plays a critical role in hippocampal-dependent learning and memory (Jones et al. 2018a). Further, our laboratory has also established that elevations of pro-inflammatory cytokines are functionally relevant to the development of enhanced fear learning following heroin administration with withdrawal (Parekh et al. 2020, 2021).

Although we did not observe membrane-dependent morphological changes using the GfaABC1D-Lck-GFP viral construct in tandem with increased GFAP volume and surface area, Lck-GFP is a structural marker that solely examines astrocyte’s structure, not reactivity. Indeed, evidence indicates that changes in astrocyte reactivity are not always associated with morphological changes, and a variety of reactivity indicators are needed to establish an inflammatory profile (Escartin et al. 2021). Our previous findings support our evaluation of an inflammatory reaction, as we have established increases in astrocyte-derived inflammatory cytokines following heroin administration and withdrawal (Parekh et al. 2020). In turn, our findings suggest that despite unchanging astrocyte morphology, astrocytes become more reactive during withdrawal.

Along with alterations in cytokine release that lead to alterations in stress responses, changes in astrocyte reactivity can also modify astrocytic interactions with adjacent neuronal elements, particularly synapses. In the present study, we did in fact observe alterations in astrocyte-neuron interactions at the 24-hour heroin withdrawal timepoint, which was indicated by increased colocalization of LCK-GFP and PSD-95 puncta. As PSD-95 serves as a marker for neural postsynaptic activity, these findings suggest that withdrawal from chronic heroin administration led to heightened astrocyte-neuron interactions, which may be related to increased fear learning through several mechanisms.

For example, increased astrocyte-neuron interactions may occur through heroin’s ability to alter astrocytic peripheral processes, especially regarding their association with neuronal synapses (Kruyer et al. 2020). Changes in these distal processes, known as leaflets, have been shown to be involved in consolidating fearful memories through neural interactions (Badia-Soteras et al. 2022; Verkhratsky et al. 2020). In turn, astrocyte-neuron interactions observed following heroin administration and withdrawal may play a role in developing the freezing behaviors in heroin withdrawal-enhanced fear learning through changes in memory encoding.

Further, heightened interactions between astrocytes and neurons may be instigated by heroin administration and withdrawal through heroin’s ability to directly activate µ-opioid receptors found on astrocytes (Nam et al. 2018). Activation of these µ-opioid receptors raises calcium levels within the cell and prompts the release of the gliotransmitter glutamate (Corkrum et al. 2019). This glutamate release initiates slow inward currents by activating neuronal N-methyl-D-aspartate (NMDA) receptors on neurons. This enhanced glutamate release and NMDA receptor activation may represent a potential mechanism for the sensitization of fear learning by heroin. This notion is reinforced by the well-established connection between PSD-95 and synaptic plasticity of glutamatergic synapses, as the NMDA receptor/PSD-95 complex exhibits one of the strongest associations within the PSD (Naisbitt et al. 1999). In turn, increased colocalization between GFAP and PSD-95 in animals exposed chronically to heroin suggests that NMDA receptors are involved in this astrocyte-neuron association.

Interestingly, we also see that we can attenuate fear learning behaviors by altering astrocyte function. We found that activation of astrocytic intracellular signaling can counteract the effects of opioid activation on astrocytes, as we observed that stimulating astroglial G_i_ signaling during heroin withdrawal significantly attenuates HW-EFL. This result could function through the inhibition of inflammatory astrocytic functioning that involves glutamatergic interactions between astrocytes and neurons. Although Experiment 3 solely examined the behavioral effect of stimulating astrocytic G_i_ signaling, we hypothesize that suppressing the increases in GFAP expression observed in Experiment 1 and 2 through activation of astrocytic G_i_ signaling underlies its ability to prevent heroin withdrawal-enhanced fear learning. The literature also suggests that corresponding changes in GFAP expression are present following astrocytic G_i_ stimulation. For example, it has been previously shown that chemogenetic stimulation of G_i_ signaling via administration of CNO diminished the LPS-induced increases in the number of GFAP‐positive astrocytes, as well as GFAP immunoreactivity in the hippocampus (Kim et al. 2021). These findings suggest that the astrocytic G_i_ pathway plays an inhibitory role in astrocyte reactivity, which supports our findings. Furthermore, stimulation of G_i_ signaling is shown to reduce LPS‐induced levels of IL-1β and TNF-α lL-1B, inflammatory cytokines that are highly colocalized with astrocytes and involved in HW-EFL (Parekh et al. 2020, 2021). In turn, activating astrocytic G_i_ signaling may inhibit elevated levels of astrocyte-derived pro-inflammatory cytokines during heroin withdrawal. Future studies should address inflammatory mediation directly by examining changes in pro-inflammatory cytokines such as IL-1β or TNF-α, which we have previously shown to be mechanistically implicated in this paradigm. Moreover, conducting additional experiments, particularly employing a distinct astrocyte construct such as a G_q_-DREADD or PMCA/CalExt, could establish a direct connection between G_i_ signaling and the observed changes, rather than attributing them to overall astrocyte modulation.

Additionally, the observed effect of stimulating G_i_ signaling in astrocytes on behavior could be mediated by the effect of G_i_-coupled signaling on calcium responses in astrocytes, as activation of endogenous G_i_-coupled signaling is shown to produce weak calcium responses (Chai et al. 2017). Weakened calcium responses could lead to decreased gliotransmitter release, including glutamate release, from astrocytes. Thus, stimulation of G_i_ signaling may prevent excess glutamatergic activation of neurons, preventing sensitization of fear-learning behaviors. Further studies can examine the effect of G_i_ pathway activation on astrocyte morphology and astrocyte-neuron interactions. Furthermore, the interconnected nature of regions such as the amygdala, striatum, and VTA raises questions about how alterations of astrocytic activation in the dentate gyrus may extend beyond localized effects (Kahn and Shohamy 2013). Future studies should aim to examine how astrocytic activation in the DG could shape broader neural circuits involved in addictive behaviors and reward mechanisms.

Overall, the findings suggest a link between enhanced astrocyte activation and heightened astrocyte-neuron interactions. These observations underscore the intricate relationship between astrocytes and neuronal elements, shedding light on the potential mechanisms by which astrocytes contribute to neuronal function, synaptic plasticity, and enhanced fear learning. These findings offer valuable insights into potential treatment targets for the development of innovative therapeutics aimed at addressing comorbid PTSD-OUD. Our findings strongly support the notion that heroin administration and withdrawal elicit a stress response, which can lead to alterations in astrocytic hippocampal synaptic plasticity and significantly impact drug-related memory formation. To better understand the precise mechanism through which hippocampal astroglial G_i_ stimulation influences heightened fear learning and heroin withdrawal, further investigations are warranted. The current study acknowledges a notable limitation concerning the lack of a direct link between the collected structural data and the observed HW-EFL effect. To address this limitation, one possible future study could utilize G_i_-DREADD to specifically investigate structural and synaptic interaction endpoints. These experiments would provide a robust connection between the findings of the first two experiments and the more functional aspects explored in experiment three. These future studies will contribute to a comprehensive understanding of the complex interplay between astrocytes, synaptic plasticity, and behavioral responses associated with PTSD-OUD, thereby paving the way for more effective therapeutic interventions.
